# Commentary: Psychometric properties of the modified Suicide Stroop Task (M-SST) in patients with suicide risk and healthy controls

**DOI:** 10.3389/fpsyg.2024.1451984

**Published:** 2024-10-10

**Authors:** Jingru Wang, Xinzhe Jin

**Affiliations:** Department of Psychology, Shaoxing University, Shaoxing, China

**Keywords:** M-SST, suicidal behavior, EEG, eye-tracking, machine learning

Suicidal behavior has always been a matter of societal concern, remaining one of the leading causes of death globally (World Health Organization, 2021). There are numerous methods for assessing suicidal behavior or ideation, among which the Suicide Stroop Task (Williams and Broadbent, 1986) has become a commonly used behavioral test. However, its psychometric properties are poor, Wilson et al. (2019) found that the scoring method for Suicide Stroop Task had low internal consistency and failed to demonstrate concurrent validity. Therefore, Gold et al. (2024) made a more detailed division of word categories. They came up with an improved version of the Stroop task and tested its psychological properties in patients with suicidal thoughts and behaviors (STB).

The task utilizes a microphone response and consists of five different word categories: neutral words (e.g., chair, fridge, towel, shower, desk, stove, door handle, bookshelf, tap), positive words (e.g., security, trust, luck, hope, strengths, ambition, confidence, friendship, honesty, passion), negative words (e.g., meanness, jealousy dispute offense, laziness, difficulty, hostility, damage, mistrust, bad luck), positive suicide-related words (e.g., suicide, sleep, silence, exit, rescue, freedom, salvation, relaxation relief, peace of mind) and negative suicide-related words (e.g., suicide, despair, fight, wish to die, pain, destruction, self-hate, end of life, leave, failure), each class consisting of 10 words. They screened positive and negative words associated with suicide from suicide online forums and showed pre-selected words to experts and clinicians in the field of suicide research to assess the emotional relevance of each word to people with STB. Each word was presented in four different colors (e.g., red, yellow, blue, and green), resulting in 40 trials per word category, and participants had to identify the color of the word. At the end of the task, participants used the suicidal Stroop survey (SSS) developed by the team to assess the word material used, obtaining participants' arousal scores for different word classes.

They found that the category of positive suicide-related words was positively correlated with self-reported suicidal ideation, but not with depression and hopelessness. Compared to healthy controls, patients were more susceptible to negative, positive suicide-related, and negative suicide-related words, with the greatest impact observed for negative suicide-related words, particularly in patients with suicidal ideation.

This study provides evidence of attentional biases toward suicide-related stimuli in patients with suicidal tendencies. Additionally, the internal reliability of the improved Stroop Task was significantly enhanced compared to the traditional version, demonstrating improved psychometric properties. Furthermore, this study is the first to conduct an additional assessment of word material used in suicide-related tasks, finding significant differences in arousal scores between STB patients and control groups across different word classes. STB patients are more affected by negative, positive suicide-related words and negative suicide-related words compared to the control group, with negative suicide-related words having the greatest impact. This approach of evaluating the word material provides valuable insights for the experimental task.

There are some points worth further discussion. First, the experimental group in this study is too limited. It would be beneficial to include suicide attempters, severely depressed patients, and participants from various age groups. For example, Baik et al. (2018) instructed severely depressed patients to perform a spatial cueing task using suicide-related and negative words as cue stimuli, finding that severely depressed patients also exhibited a suicidal attention bias. Richard-Devantoy et al. conducted a cross-sectional study using an improved Stroop task and found no between-group differences in interference scores between suicide attempters and emotional disorder patients. However a meta-analysis revealed a significant attention bias toward suicide-related words in suicide attempters compared to emotional disorder patients (Richard-Devantoy et al., 2016). Liu et al. (2022) conducted a systematic review and meta-analysis of pre-adolescent children's suicidal ideation and behavior, finding lifetime prevalence rates of suicidal ideation, suicide attempts, and non-suicidal self-harm of 15.1%, 2.6%, and 6.2%, respectively, and indicating that ~17% of children with suicidal ideation transition to suicide attempts. These studies indicate that suicidal attention bias may occur in different age groups, not just in young people, and not only in individuals with suicidal behavior or ideation but also in severely depressed patients. Therefore, selecting only participants with suicidal ideation or behavior for the improved Stroop Task introduces bias and limits the generalizability of the findings to other populations.

In addition, eye-tracking and electroencephalography (EEG) devices can be helpful in measuring increase reaction time and accuracy during task execution. Eye-tracking devices can effectively quantify participants' dwell time on words and their viewing strategies, as individuals with suicidal thoughts or behaviors tend to spend more time on negative and positive suicide-related words. Correspondingly, their brain activity might exhibit stronger activation. Tavakoli et al. used EEG to measure suicidal attention bias in adolescents with a history of suicide attempts during an emotional Stroop task, observing a dual-peak P3 component in both the suicide attempt and healthy control groups. Compared to the control group, both early and late P3 amplitudes were significantly reduced in the suicide attempt group, and the late P3 latency was significantly prolonged (Tavakoli et al., 2021). Toh et al. used eye-tracking devices to investigate attentional bias in patients with body dysmorphic disorder using an emotional Stroop task, finding significant Stroop interference with body dysmorphic negative words. Additionally, analysis of scanning patterns and gaze heat maps revealed that the experimental group employed disorganized viewing strategies, avoiding certain disease-related words and focusing visual attention on non-prominent areas (Toh et al., 2017). Tsypes et al. assessed children's attentional bias toward facial emotional expressions using a modified dot-detection task and eye-tracking device, measuring gaze location and duration in children with suicidal ideation (SI). Children with a history of SI were found to gaze at fearful faces for significantly longer (Tsypes et al., 2017). As a result, the reaction time can be measured more accurately.

Therefore, the use of eye-tracking and EEG devices can effectively reflect the suicidal attention bias in patients with suicidal behavior or intent. Eye-tracking devices can measure participants' gaze duration and visual attention, while EEG can visually indicate the level of activation in response to stimuli, providing a more intuitive indication of whether patients exhibit suicidal attention bias.

Furthermore, this study found a significant correlation between negation words and negative suicide-related words, suggesting some commonalities between these two categories of words. This might be due to subjective selection of words by the researchers or the use of questionnaires for scoring, which introduces certain biases. Therefore, improvements can be made in the word selection process. For instance, computer algorithms could be employed to select words from suicide forums, filtering for high-frequency terms. Subsequently, machine learning (ML) and natural language processing (NLP) technologies could be utilized for statistical tasks such as text classification or emotion mining, leveraging large corpora and sophisticated learning methods to classify negation words and negative suicide-related words (Le Glaz et al., 2021). Questionnaires could then be used to score the words. Guan et al. (2015) identified Chinese users with a high likelihood of suicide by extracting language features from internet data using simple logistic regression (SLR) and random forest (RF) models. Similarly, Doan et al. (2017) analyzed tweets containing keywords like “stress” and “relaxation,” employing support vector machines (SVM) and naive Bayes (NB) for classification. Thus, using models for word classification may offer a more objective approach.

In conclusion, this study is highly significant for patients with suicidal behavior or ideation. The emergence of this task can effectively assess the suicidal attention bias in STB patients, providing an assessment tool for patient treatment and suggesting a promising direction for the future. However, there are some shortcomings that can be addressed in subsequent studies, such as including a larger and more diverse participant group, using more standardized methods for word selection, and employing more accurate response techniques. Implementing these measures can enhance the ecological validity of the improved Suicide Stroop Task and enable its generalization to different populations.
